# Choroidal thickness on optical coherence tomography as a longitudinal predictor of visual outcomes in intermediate uveitis

**DOI:** 10.1038/s41598-026-58318-z

**Published:** 2026-06-17

**Authors:** Suchitra Biswal, Pradeep Sagar, Marie D. Just, Nicholas R. Merten, Moritz Berger, Jan H. Terheyden, Matthias Schmid, Frank G. Holz, Robert P. Finger, Maximilian W. M. Wintergerst

**Affiliations:** 1 Department of Medical Retina and Uvea, Sankara Eye Hospital, Shivamogga, India; 2 Department of Ophthalmology, University Hospital Bonn, University of Bonn, Bonn, Germany; 3 Core Facility Biostatistics, Medical Faculty Mannheim, Central Institute of Mental Health, Heidelberg University, Mannheim, Germany; 4 Department of Medical Biometry, Informatics and Epidemiology, University Hospital Bonn, University of Bonn, Bonn, Germany; 5 Department of Ophthalmology, Medical Faculty Mannheim, Heidelberg University, Mannheim, Germany; 6 Augenzentrum Grischun, Chur, Switzerland; 7 Department of Ophthalmology, University Hospital Bonn, Ernst-Abbe-Straße 2 |, 53127 Bonn, Germany

**Keywords:** Intermediate uveitis, Optical coherence tomography, OCT, Longitudinal, Biomarkers, Diseases, Medical research

## Abstract

To assess longitudinal changes in subfoveal choroidal thickness (SFCT) and mean choroidal thickness (MCT) in intermediate uveitis, eyes with at least one follow-up visit were included. These eyes were stratified into clinically worsened, stable, or improved based on changes in clinical parameters including Standardization of Uveitis Nomenclature (SUN) classification, to evaluate their prognostic value for future best-corrected visual acuity (BCVA) and central retinal thickness (CRT). Spectral domain optical coherence tomography (Heidelberg Engineering, Germany) was used to image the central macula. SFCT, MCT, and CRT were measured manually within the central 1 mm. Mixed-effects regression analysis controlling for age and sex was used to evaluate the prognostic value of SFCT and MCT regarding future BCVA and CRT. A total of 91 eyes from 52 patients were included in the analysis. While 12 eyes worsened, 62 remained stable, and 17 improved. Choroidal thickness remained stable over time, with no significant differences in SFCT or MCT change between clinical groups (*p* > 0.5 for all). When controlling for age and sex, both the baseline SFCT (estimate = -0.35 × 10^−^ ³ logMAR per µm, *p* = 0.040) and MCT (estimate = -0.42 × 10^−^³ logMAR per µm, *p* = 0.018) were prognostic for future BCVA. MCT, but not SFCT, was significantly associated with future CRT (estimate = -0.15 μm per µm, *p* = 0.010 vs. -0.13 μm per µm, *p* = 0.140). Choroidal thickness in terms of baseline SFCT and MCT is prognostic of future BCVA and could serve as a prognostic structural biomarker for intermediate uveitis.

## Introduction

Intermediate uveitis is a subset of uveitis in which the inflammation is mainly focused on the vitreous^1^. It accounts for 1.2% to 22% of patients with uveitis in the western population^2^. The chronic course of the disease can lead to complications including cystoid macular edema, retinal detachment, retinal neovascularization, glaucoma, and blindness^3^. Chronic intermediate uveitis requires long term immunosuppressive therapy and exacerbation of inflammation during the course of treatment may necessitate additional immunosuppression^4^. Identification of the presence or absence of active inflammation is primarily based on the clinical judgement. Clinical features of acute exacerbation include worsening of visual acuity, presence of anterior chamber cells, and an increase in floaters, or vitreous cells. Fundus fluorescein angiography enables visualization of leakage from the optic nerve head and retinal vessels during the active phase; however, its invasive nature represents a relevant limitation^5^. In intermediate uveitis, recurrent macular edema is a major driver of long-term decline in best-corrected visual acuity (BCVA). However, as with many rare diseases, there is a lack of high-level evidence regarding monitoring and outcome, partly due to a dearth of reliable and objective quantitative endpoints for clinical trials^6^.

Optical coherence tomography (OCT) is a non-invasive tool and is helpful in detection of macular edema in eyes with intermediate uveitis^7,8^. Though the predominant site of inflammation in intermediate uveitis is the vitreous, only a few studies have evaluated an association between choroidal thickness and disease activity. A recent case control study noted no difference in choroidal thickness in eyes with intermediate uveitis and healthy controls^9^. In a retrospective study of 148 eyes with inactive uveitis and 98 healthy eyes, eyes with uveitis were noted to have thinner choroid compared to normal subjects^10^. In a study evaluating the longitudinal changes in choroidal parameters in eyes with active intermediate uveitis, it was concluded that the choroidal vascular index (CVI) and the luminal area of choroidal vessels increased from baseline to resolution and the stromal area reduced from baseline to resolution^11^. In our previous work on the role of OCT angiography in intermediate uveitis, we identified that the non-perfusion areas in the choriocapillaris layer increased in eyes with worsening of inflammation compared to eyes with improvement and eyes that were stable^8,12^.

Considering the limited data available in the literature, this retrospective study was conducted to evaluate the longitudinal changes in choroidal thickness during the course of disease and its impact on central retinal thickness and BCVA.

## Methods

This is a retrospective study of cases diagnosed with intermediate uveitis according to Standardization of Uveitis Nomenclature (SUN)^1^ at the Department of Ophthalmology, University Hospital Bonn, Germany. The present study was conducted in the same retrospective cohort as a previously published longitudinal study on OCT angiography parameters in intermediate uveitis from our group^12^. Cases with at least one follow-up visit were included. The study was approved by the institutional ethics committee of the University of Bonn (approval ID 548/20). Informed consent was obtained from all subjects and/or their legal guardian(s) before enrollment. The study adhered to the tenets of the declaration of Helsinki. Cases with poor OCT image quality in which the chorioscleral interface was not identifiable, with diabetes mellitus, and with retinal diseases other than non-infectious intermediate uveitis were excluded from the study. Additionally, eyes with outer retinal atrophy, defined as hypertransmission or disruption of the outer retinal bands, were excluded if located at the fovea or extending over more than 100 μm.

Demographic data were collected from medical records. BCVA was converted into logMAR. Inflammation was graded based on the presence of cells in the anterior chamber (SUN working group grading)^1^, vitreous cells (Nussenblatt et al.)^13^, vitreous haze (scale for vitreous haze grading in uveitis)^14^, snowbanking, snowballs and vasculitis.

**Fig. 1:**
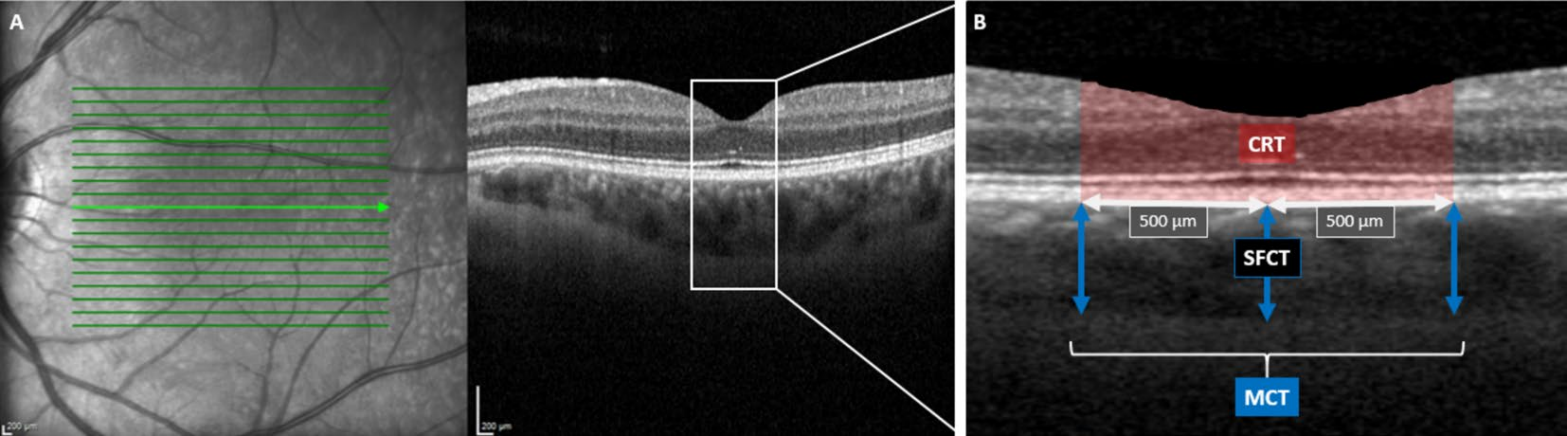
Representative optical coherence tomography scan of measurements. **(A)** En face infrared image (left) with exemplary scan through the fovea (right) in 1:1 pixel. **(B)** Magnification of the white square displayed in 1:1 μm with measurement of subfoveal choroidal thickness (SFCT) and choroidal thickness nasal and temporal to the foveal center; all three measurements represent the mean choroidal thickness (MCT); choroidal thickness is measured from the outer boundary of retinal pigment epithelium-Bruch’s membrane complex to chorioscleral interface; central retinal thickness (CRT) is measured within a 500 μm radius around the fovea (ETDRS central 1 mm circle, not shown here).

Spectral domain OCT (Heidelberg Spectralis, Heidelberg Engineering, Heidelberg, Germany) was used to image the central macula (30° macula-centered horizontal linear scan). Macular edema was classified as absent, present with normal foveal contour, and present with altered foveal contour. Central retinal thickness (CRT) was measured within the central 1 mm circle of the early treatment of diabetic retinopathy study (ETDRS) grid. Subfoveal choroidal thickness and juxtafoveal choroidal thickness at 500 (495–505) µm nasal to and temporal to the foveal center in the horizontal meridian was measured by two independent ophthalmologists. Choroidal thickness was measured manually using the caliper available in the software, perpendicular to the retinal pigment epithelium (RPE)-Bruch’s membrane complex to the chorioscleral interface (Fig. 1).

Eyes were classified into three groups: “worsened”, “stable” and “improved” based on the following changes in clinical features including SUN activity classification^1^ from first visit to follow-up visit: two step worsening or improvement in anterior chamber cell grading, vitreous cell grading, vitreous haze grading; appearance or disappearance of snowbanking, snowballs, and vasculitis; change in macular edema classification; change in CRT by 100 μm or more. If more than two visits were available, two visits with pronounced changes in above-mentioned clinical features were considered for analysis.

### Statistical analysis

Statistical analyses were performed with R (R: A Language and Environment for Statistical Computing, version 4.5.0, R Core Team, R Foundation for Statistical Computing, Vienna, Austria, 2025). Interobserver agreement was assessed by calculating the intraclass correlation coefficient (ICC) model “two-way mixed”, type “consistency”, unit “single”^15,16^. Friedman test for repeated measures was used for the comparison of OCT parameter changes between the three clinical groups. The comparison of OCT parameter changes (SFCT, MCT at 3 regions: subfoveal, 500 μm nasal to the foveal centre, 500 μm temporal to the foveal centre, and CRT; see Fig. 1) and clinical groups was additionally adjusted for age and sex at baseline and follow-up as possible confounders using a multivariable linear mixed-effects regression model (including a random intercept for each patient).

Furthermore, multivariable linear mixed-effects regression analyses using all available visits (as defined in the previous paragraph) were performed in order to evaluate the prognostic value of choroidal thickness for BCVA and CRT. Future BCVA and future CRT were defined as dependent and current choroidal thickness, current BCVA and current CRT as independent variables. P-values < 0.05 were considered statistically significant.

## Results

### Demographic and clinical characteristics

A total of 91 eyes of 52 patients were included in the analysis and divided into the three groups as outlined above. Sixteen eyes were excluded from statistical analyses based on the above-mentioned exclusion criteria, of which three were excluded due to poor image quality. The clinically worsened group included 12 eyes, the clinically stable group included 62 eyes, and the clinically improved group included 17 eyes. Baseline and follow-up characteristics of the sample are reported in Table 1. The mean age at baseline was 48 ± 18 years in the worsened, 47 ± 18 years in the stable, and 41 ± 21 years in the improved group; 58%, 40%, and 71% of eyes belonged to male patients, respectively. Idiopathic non-pars planitis was the most common intermediate uveitis aetiology across all three groups (50%, 69%, and 65% in the worsened, stable, and improved groups, respectively). At baseline, treatment was present in 67%, 37%, and 71% of eyes in the worsened, stable, and improved groups, respectively. At baseline, BCVA was 0.25 ± 0.23 logMAR units in the worsened group, 0.06 ± 0.18 logMAR units in the stable group, and 0.32 ± 0.25 logMAR units in the improved group. No statistically significant differences in spherical refractive error were observed among the three groups. At baseline, CRT was 278.33 ± 68.45 μm, 299.82 ± 58.67 μm, and 448.18 ± 116.96 μm, respectively (Table 1). At baseline, macular edema was absent in 83%, 90%, and 0% of eyes in the worsened, stable, and improved groups, respectively. The improved group showed the highest proportion of macular edema at baseline (100%), with resolution in the majority of eyes at follow-up. Non-foveal retinal atrophy at baseline was present in 3 eyes (25%) in the worsened group, 1 eye (2%) in the stable group, and none in the improved group. The mean follow-up time across all groups was 296 ± 273 days.

**Table 1:** Baseline and follow-up characteristics of the study sample stratified by clinical course. Values are presented as mean ± SD (range) or n (%). BCVA: Best-corrected visual acuity; dpt: diopters; CRT: Central retinal thickness; SFCT: Subfoveal choroidal thickness; MCT: Mean choroidal thickness.

	Clinically Worsened Group (*n* = 12 eyes)		Clinically Stable Group (*n* = 62 eyes)		Clinically Improved Group (*n* = 17 eyes)	
	Baseline	Follow-up	Baseline	Follow-up	Baseline	Follow-up
Age at baseline (years)	48 ± 18 (26–80)		47 ± 18 (17–90)		41 ± 21 (17–90)	
Sex (male)	7 (58%)		25 (40%)		12 (71%)	
**Intermediate uveitis aetiology**						
Idiopathic (pars planitis)	3 (25%)		7 (11%)		2 (12%)	
Idiopathic (non-pars planitis)	6 (50%)		43 (69%)		11 (65%)	
Systemic	3 (25%)		10 (16%)		4 (24%)	
Drug-induced	0 (0%)		2 (3%)		0 (0%)	
**Presence of treatment at baseline**						
Total	8 (67%)		23 (37%)		12 (71%)	
Topical steroids	1 (8%)		6 (10%)		4 (24%)	
Periocular steroids	0 (0%)		0 (0%)		0 (0%)	
Intraocular steroids	3 (25%)		5 (8%)		3 (18%)	
Systemic steroids	2 (17%)		4 (7%)		5 (29%)	
Immunosuppression	4 (33%)		9 (15%)		4 (24%)	
Biologicals	2 (17%)		5 (8%)		5 (29%)	
Spherical refractive error (dpt)	0.10 ± 1.11		0.76 ± 2.34		−0.06 ± 1.77	
Lens status (phakic)	8 (67%)	5 (42%)	47 (76%)	47 (76%)	13 (76%)	12 (71%)
BCVA (logMAR)	0.25 ± 0.23	0.44 ± 0.31	0.06 ± 0.18	0.07 ± 0.18	0.32 ± 0.25	0.05 ± 0.08
CRT (µm)	278.33 ± 68.45	398.08 ± 166.05	299.82 ± 58.67	298.65 ± 53.83	448.18 ± 116.96	296.47 ± 30.19
SFCT (µm)	275.83 ± 95.84 (146–449)	262.08 ± 94.43 (141–436)	292.79 ± 64.95 (183–467)	291.75 ± 75.47 (131–513)	331.00 ± 97.56 (165–535)	333.38 ± 62.28 (183–429)
MCT (µm)	268.53 ± 95.87 (131–432)	256.50 ± 91.13 (127–412)	281.30 ± 59.64 (177–433)	282.98 ± 70.14 (132–484)	321.76 ± 95.24 (162–521)	318.73 ± 58.35 (184–419)
**Macular edema**						
No macular edema	10 (83%)	2 (17%)	56 (90%)	57 (92%)	0 (0%)	16 (94%)
Macular edema (normal foveal contour)	2 (17%)	1 (8%)	4 (7%)	3 (5%)	8 (47%)	1 (6%)
Macular edema (altered foveal contour)	0 (0%)	9 (75%)	2 (3%)	2 (3%)	9 (53%)	0 (0%)
Retinal atrophy at baseline†	3 (25%)		1 (2%)		0 (0%)	
Duration of uveitis at baseline (months)	85 ± 57		99 ± 64		73 ± 55	
Time between baseline and follow-up (days)	327 ± 265		264 ± 229		388 ± 326	

† Retinal atrophy defined as hypertransmission or disruption of the outer retinal bands (< 100 μm, non-foveal).

### Longitudinal changes of choroidal thickness

**Fig. 2:**
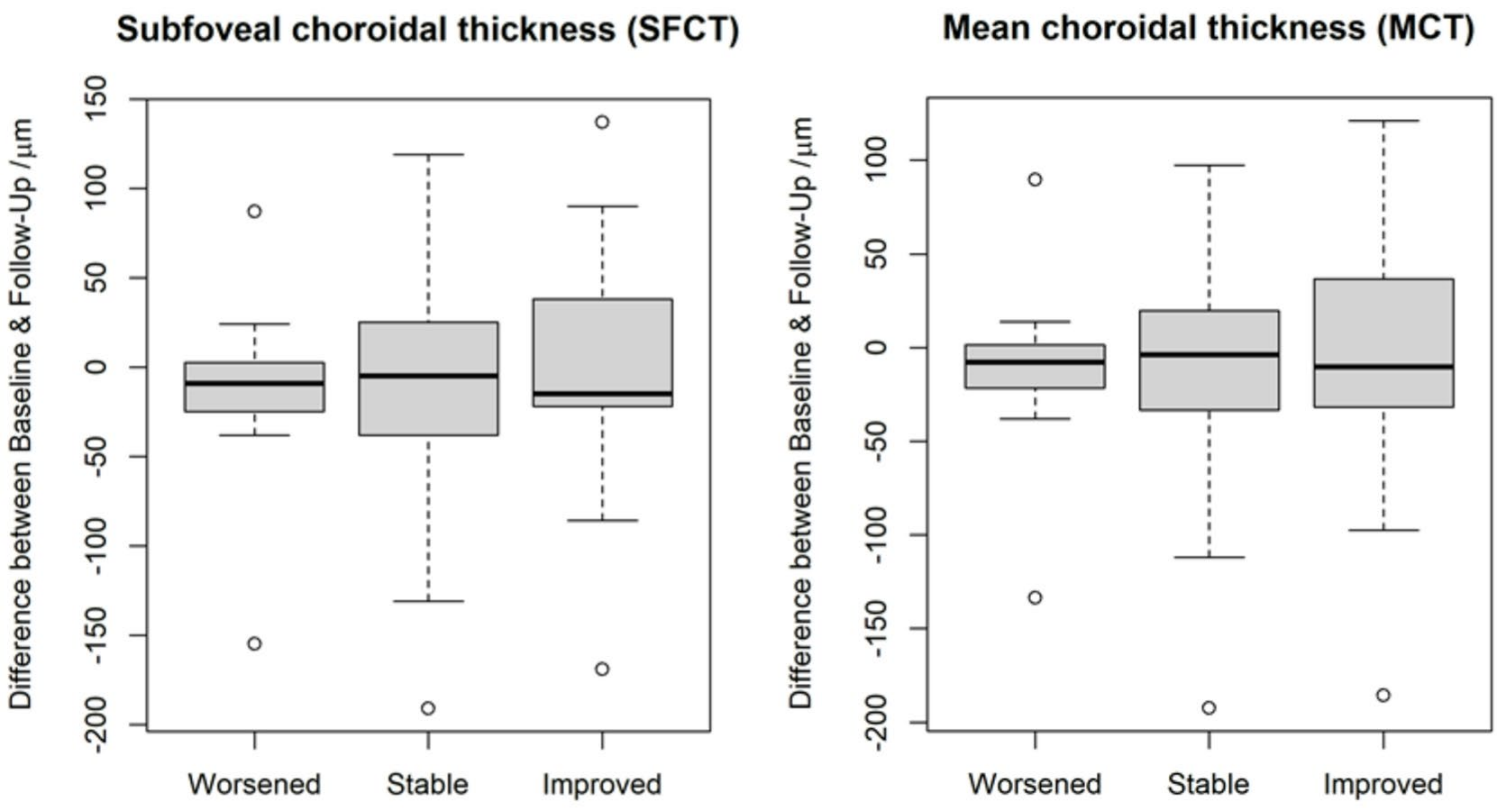
Change in subfoveal and mean choroidal thickness. Longitudinal analysis of absolute difference in subfoveal and mean choroidal thickness between baseline and follow-up on optical coherence tomography in eyes with intermediate uveitis. Change in choroidal thickness is compared between the three groups “worsened”, “stable” and “improved”, defined by their clinical development. Outliers were defined as values over 1.5 interquartile range below the first quartile or above the third quartile.

A good interobserver agreement was noted in the measurement of choroidal thickness between the two observers (SFCT: ICC = 0.79, 0.70 < ICC < 0.85; CT 500 μm nasal to the foveal centre: ICC = 0.76, 0.66 < ICC < 0.83; CT 500 μm temporal to the foveal centre: ICC = 0.81, 0.72 < ICC < 0.87). The SFCT and MCT at baseline and follow-up in the three groups are presented in Table 1. SFCT and MCT at baseline were highest in the improved group (331.00 ± 97.56 μm and 321.76 ± 95.24 μm), intermediate in the stable group (292.79 ± 64.95 μm and 281.30 ± 59.64 μm), and lowest in the worsened group (275.83 ± 95.84 μm and 268.53 ± 95.87 μm). However, longitudinal analysis of change in SFCT and MCT between baseline and follow-up revealed no statistically significant differences between the three clinical outcome groups (all *p* > 0.5; Fig. 2).

In mixed-effects regression models adjusted for age and sex, clinically worsened eyes showed a non-significant mean reduction in SFCT (estimate = −7.02 μm, *p* = 0.75) and MCT (estimate = −5.13 μm, *p* = 0.80) compared to clinically stable eyes. Clinically improved eyes also showed no significant change in SFCT (estimate = −2.29 μm, *p* = 0.91) or MCT (estimate = −12.02 μm, *p* = 0.50) compared to the clinically stable group. Age and sex were not significantly associated with changes in choroidal thickness (all *p* > 0.5). Collectively, these findings suggest that choroidal thickness remains relatively stable over time regardless of clinical disease course (Table 2).

**Table 2:** Change in subfoveal and mean choroidal thickness. Confounding analysis. Multivariable regression analysis using linear mixed-effects models including a random intercept for each patient (worsened and improved: compared to stable group). SFCT: Subfoveal choroidal thickness; MCT: Mean choroidal thickness.

	Change in SFCT			Change in MCT		
	Estimate	Std. Error	*p*	Estimate	Std. Error	*p*
Intercept	39.53	114.27	0.73	18.07	105.64	0.86
Clinically worsened	−7.02	22.50	0.75	−5.13	20.80	0.80
Clinically improved	−2.29	19.40	0.91	−12.02	17.93	0.50
Age	0.17	0.38	0.65	0.21	0.34	0.54
Sex	0.50	14.06	0.97	−2.05	12.99	0.87

### Prognostic value of choroidal thickness

Higher baseline SFCT and MCT were significantly associated with better BCVA at follow-up (Table 3). For SFCT, the regression coefficient was − 0.35 × 10^−^³ logMAR per µm (*p* = 0.040), indicating that thicker choroid at baseline tended towards better visual outcomes. MCT showed a comparable and slightly stronger effect (estimate = −0.42 × 10^−^³ logMAR per µm, *p* = 0.018). Baseline BCVA was also a strong predictor in both models (*p* = 0.008 and *p* = 0.012, respectively).

**Table 3:** Prognostic analysis of optical coherence tomography parameters for future BCVA. Multivariable regression analyses using linear mixed models for future BCVA as dependent variable including a random intercept for each patient. Independent variables: Baseline SFCT, Baseline MCT, Baseline BCVA. SFCT: Subfoveal choroidal thickness; MCT: Mean choroidal thickness; BCVA: Best-corrected visual acuity.

	Future BCVA			Future BCVA		
	Estimate	Std. Error	*p*	Estimate	Std. Error	*p*
Intercept	0.35	0.11	0.002	0.37	0.11	0.001
Baseline SFCT/Baseline MCT (x 10^− 3^ logMAR units)	−0.35	0.17	0.040	−0.42	0.18	0.018
Baseline BCVA (logMAR units)	0.15	0.06	0.008	0.14	0.05	0.012

### Prediction of future central retinal thickness

Baseline MCT, but not SFCT, was significantly associated with future central retinal thickness (CRT) (Table 4). The estimate for MCT was − 0.15 μm per µm (*p* = 0.010), suggesting that thicker choroid at baseline was associated with thinner CRT at follow-up, potentially reflecting less macular edema. In contrast, the association between SFCT and future CRT did not reach statistical significance (estimate = −0.13 μm per µm, *p* = 0.14). In both models, baseline CRT remained a strong predictor of future CRT (*p* = 0.001).

**Table 4:** Prognostic analysis of optical coherence tomography parameters for future CRT. Multivariable regression analyses using linear mixed models for future CRT as dependent variable including a random intercept for each patient. Independent variables: Baseline SFCT, Baseline MCT, Baseline CRT. SFCT: Subfoveal choroidal thickness; MCT: Mean choroidal thickness; CRT: Central retinal thickness.

	Future CRT			Future CRT		
	Estimate	Std. Error	*p*	Estimate	Std. Error	*p*
Intercept	262.27	81.19	0.001	269.11	81.44	0.001
Baseline SFCT/Baseline MCT	−0.13	0.09	0.14	−0.15	0.09	0.010
Baseline CRT	0.23	0.07	0.001	0.23	0.07	0.001

## Discussion

Our longitudinal analysis indicates that choroidal thickness (both subfoveal and mean) remains relatively stable over time in intermediate uveitis, irrespective of the clinical course, including stable disease, improvement, or worsening. This supports the view that choroidal thickness is not an indicator of disease activity and is unlikely to serve as a structural endpoint. Importantly, using a longitudinal baseline-to-follow-up design, the present study indicates that higher baseline choroidal thickness is prognostic of better future BCVA, irrespective of the clinical course. This would suggest that higher choroidal thickness may be protective against visual loss in eyes with intermediate uveitis.

It is a well-established fact that choroid is thicker in active choroiditis^17^, however whether intermediate uveitis has an effect on choroid is not well understood. To the best of our knowledge, one study has evaluated the longitudinal changes in choroidal parameters during the course of disease in eyes with intermediate uveitis. In a retrospective study of 38 eyes with active intermediate uveitis and 30 eyes with quiescent intermediate uveitis, Kongwattananon et al.^11^noted no significant change in subfoveal choroidal thickness from baseline to follow up in eyes with active and quiescent intermediate uveitis which is consistent with our observations that choroidal thickness is not an indicator of disease activity in intermediate uveitis. In addition to measurement of subfoveal choroidal thickness, Kongwattananon et al. evaluated CVI, luminal area, stromal area. They noted an increase in CVI, luminal area and a decrease in stromal area from baseline with resolution of inflammation on follow-up indicating that choroid would be affected in intermediate uveitis with differential effect on vascular lumen and stroma^11^. Our study indicates that choroidal thickness is not an indicator of inflammatory activity in intermediate uveitis, but this does not preclude an effect on vascular lumen and stromal volume or area.

Our observation indicates that higher baseline choroidal thickness may be prognostic for better future functional outcomes irrespective of clinical course. Importantly, choroidal thickness remained unchanged across clinical groups, suggesting that its relationship to future vision is not a function of inflammation-mediated effects such as vitreous haze. Moreover, baseline SFCT did not predict future CRT, whereas MCT showed a significant association with future CRT (*p* = 0.010), suggesting that the relationship between choroidal thickness and visual acuity may at least in part be independent of macular edema. A possible explanation is that intermediate uveitis is not confined to peripheral vascular inflammation alone, but may also involve retinal/choroidal vasculitis, leading to structural and functional retinal changes independent of macular edema^18^. While the observed effect sizes were modest (SFCT: −0.35 × 10⁻³ logMAR per µm; MCT: −0.42 × 10⁻³ logMAR per µm), they reached statistical significance after adjustment for age, sex, and baseline BCVA. We acknowledge that the absolute magnitude of these associations may not translate directly into large individual-level differences in visual acuity, and confirmation in larger prospective cohorts is warranted. Nevertheless, in the context of a rare disease with few validated prognostic markers, even modest associations with a routinely measurable parameter may have meaningful implications for patient stratification and future trial design.

To the best of our knowledge, no other studies have evaluated the correlation between choroidal thickness and visual acuity in eyes with intermediate uveitis. A study evaluating the association between visual acuity and choroidal thickness in young individuals noted significant association between worse BCVA and thinner choroid^19^ which may indicate that higher choroidal thickness may be protective to overlying retina. However, similar results are noted only in myopic eyes^20,21^ in which atrophic maculopathy is associated with thin choroid and poor BCVA. A study evaluating the correlation between choroidal thickness and visual acuity in diabetic macular edema, noted no significant correlation^22^. Based on these points, though our study indicated a prognostic value of baseline choroidal thickness, the underlying biological mechanism remains unclear.

The present study was performed in the same retrospective cohort as a previously published study evaluating OCT angiography parameters in intermediate uveitis^12^. While the use of an established, well-characterised cohort strengthens comparability between studies, findings should be interpreted in this context and require validation in independent prospective cohorts. A notable strength of this study is the focus on choroidal thickness, a parameter that can be measured on widely available spectral-domain OCT devices without specialized post-processing software, rendering it a potentially accessible and cost-effective prognostic biomarker for routine clinical practice. The retrospective design introduces potential selection bias and limits full control over treatment variability and follow-up intervals. Additional limitations include the relatively small sample size of the “worsened” and “improved” subgroups. In the context of a relatively rare disease such as intermediate uveitis, the final cohort of 52 patients (91 eyes) represents a clinically meaningful and methodologically robust longitudinal dataset. Stringent exclusion criteria applied to ensure adequate image quality, longitudinal follow-up, and complete clinical characterization inevitably reduced sample size but strengthened internal validity. Importantly, the longitudinal baseline-to-follow-up design with adjustment for baseline BCVA enhances statistical efficiency and allows estimation of clinically relevant prognostic associations despite a moderate cohort size. While small effect sizes may remain undetected, the present study provides sufficient precision to identify and estimate moderate prognostic relationships under real-world conditions. Moreover, our assessment was limited to a single structural choroidal parameter (thickness), which may not capture the full complexity of choroidal changes. However, as outlined above, this choice was deliberate, given the clinical accessibility of choroidal thickness measurements. More comprehensive imaging (e.g., CVI or flow-based metrics) could provide additional insight. Furthermore, we did not specifically assess for the pachychoroid phenotype (characterized by dilated Haller layer vessels with attenuation of Sattler’s layer and choriocapillaris) while measuring choroidal thickness. Although cases of central serous chorioretinopathy (CSC), pachychoroid neovasculopathy (PNV), and polypoidal choroidal vasculopathy (PCV) were excluded, it is possible that eyes with pachychoroid pigment epitheliopathy or asymptomatic pachychoroid were inadvertently included, which represents a limitation of our study. Differences in uveitis aetiology and treatment presence at baseline were observed across clinical groups, and neither variable was included as a covariate in the regression models, representing a potential source of confounding. Notably, the improved group had the highest proportion of treated eyes (71%) compared to the stable group (37%). To the best of our knowledge, no studies have specifically investigated the effect of immunosuppressive or biological therapy on choroidal thickness in intermediate uveitis. It is therefore conceivable that treatment differences between groups may have contributed to the observed baseline choroidal thickness differences or influenced the prognostic associations identified. Similarly, although eyes with foveal or extensive outer retinal atrophy (> 100 μm) were excluded, a small number of eyes with residual non-foveal atrophy was retained, with a higher proportion in the worsened group (25%) compared to the stable (2%) and improved (0%) groups. Finally, cataract progression was not accounted for. Taken together, these uncontrolled variables may have influenced the observed associations and should be addressed in future prospective studies.

In conclusion, choroidal thickness may serve as a prognostic marker of future visual acuity in intermediate uveitis; however, it may not be a structural endpoint or adequate activity marker. Given its clinical accessibility and wide availability, choroidal thickness represents a promising candidate prognostic biomarker that warrants further investigation in larger, prospective studies. These findings are hypothesis-generating and warrant further investigation into the potential of choroidal imaging as a clinically applicable tool in the management and stratification of intermediate uveitis.

### Statement

During the preparation of this work the authors used generative AI tools, namely ChatGPT (OpenAI, San Francisco, CA, USA) and Claude (Anthropic, San Francisco, CA, USA), to improve readability and language. After using this tool, the authors reviewed and edited the content as needed and take full responsibility for the content of the published article.

## Data Availability

Data is available upon reasonable request. For inquiries, please contact the corresponding author.
